# From Seedling Architecture to Grain: Linking Root and Shoot Traits with Yield Potential for Climate-Resilient Wheat Breeding

**DOI:** 10.3390/plants15192900

**Published:** 2026-09-22

**Authors:** Amal Gamal Mohamed, Amira M. I. Mourad, Atif Aboelwafa, Helmy M. Youssef, Mohammed A. Sayed

**Affiliations:** 1 Agronomy Department, Faculty of Agriculture, Assiut University, Assiut 71526, Egypt; amlgamal8899@gmail.com (A.G.M.); amira_mourad@aun.edu.eg (A.M.I.M.); atif@aun.edu.eg (A.A.); 2 Institute of Agricultural and Nutritional Sciences, Martin-Luther-University Halle-Wittenberg, Betty-Heimann-Str. 3, D-06120 Halle (Saale), Germany; 3 Center for Converging Sciences and Emerging Technologies (CoSET), Benha National University, Al Obour 13518, Egypt

**Keywords:** spring bread wheat, root system architecture, path coefficient analysis, multivariate biplots, MGIDI ideotype

## Abstract

Improving wheat for climate change requires understanding how seedling root systems affect adult plant performance. We evaluated 116 spring bread wheat (*Triticum aestivum* L.) genotypes across 25 traits under non-limiting moisture conditions. Analysis of variance revealed significant genotypic variation (*p* < 0.01) for all traits. High broad-sense heritability (H^2^ up to 99.81%) and close alignment between genotypic (GCV) and phenotypic (PCV) coefficients of variation indicated substantial genotypic differentiation among the evaluated accessions under these experimental conditions. A 21-predictor path analysis (R^2^ = 0.80) identified the number of spikes per plant (NSP; direct effect = 0.51) and heading date (HD; −0.32) as the primary positive and negative determinants of grain yield per plant (GYP), respectively. Hierarchical cluster analysis (HCA) and principal component analysis (PCA) resolved four distinct clusters, revealing a functional trade-off between early vegetative vigor and mature yield: elite cultivars and accessions (Cluster 1) combined compact shoots, a high root-to-shoot ratio (RSR = 1.55), and superior yield (GYP = 11.76 g), whereas excessive seedling biomass was associated with yield penalties. Deep seminal-root angle (DRA) established a steep spatial distribution. The multi-trait genotype-ideotype distance index (MGIDI) identified 17 elite genotypes (e.g., G024, G020, G106, Sids14, and Gemiza11), offering an expected +32.3% genetic gain in GYP. These constitutive baseline architectural criteria provide reliable early-stage tools for breeding climate-adapted wheat.

## 1. Introduction

Bread wheat (*Triticum aestivum* L.) is one of the most essential crops, contributing about 20% of calories and one-third of daily caloric intake in Egypt [1,2,3,4]. It accounts for about 10% of the country’s total agricultural production value [4]. As a key national strategic crop, improving wheat productivity is essential for ensuring food security in Egypt and minimizing reliance on imports, which are further strained by climate change and economic challenges [5,6,7]. Sustaining yield potential under changing climatic conditions requires breeding strategies that focus on non-traditional traits, particularly root and shoot architectural characteristics that govern resource acquisition efficiency. Therefore, understanding and developing these traits is key to increasing wheat productivity [8,9,10,11,12].

Root system architecture (RSA) in wheat, including seminal root number, growth angles, and length, exhibits genetic variation and developmental plasticity influenced by genotype-by-environment (G × E) interactions [13,14]. Modern varieties tend to have reduced RSA variation, favoring deeper, less branched roots that enhance yield; however, beneficial variation still exists within contemporary wheat populations [15,16,17]. Improved productivity is associated with an increased number of embryonic roots and narrower growth angles, which confer drought tolerance, while thinner, shallower roots improve nitrogen uptake efficiency [9,12,18,19,20]. Therefore, exploiting this variation in root traits remains a cornerstone of wheat breeding programs aimed at developing varieties adapted to the challenges posed by a changing climate.

Despite the crucial role of RSA in determining plant efficiency, evaluating roots in the field remains a laborious, costly, and time-consuming process. Additionally, it is destructive to the plant, making it difficult to study large numbers of genotypes [21,22,23]. To overcome these challenges, modern wheat breeding programs have focused on linking root characteristics at the seedling stage to final yield and its attributes in the field. Several studies (e.g., Atkinson et al. [24]; Richard et al. [25]) have demonstrated strong genetic and physiological correlations between early seminal root traits, including seminal root number, root angle, and growth vigor, and subsequent root distribution depth. These traits can enhance nutrient and water uptake, ultimately contributing to improved yield. Therefore, high-throughput phenotyping at the seedling stage enables rapid and efficient screening of hundreds of genotypes for root traits. However, a critical knowledge gap remains regarding whether early seminal root architecture can reliably predict the performance of mature plants under field conditions, thereby limiting its direct application as a selection criterion in breeding programs. Středa et al. [26] highlighted that root traits are significantly influenced by environmental factors, resulting in variability in their expression across different growth conditions. Consequently, the root architecture of early seedlings should be regarded as a preliminary indication of root development rather than a definitive portrayal of the mature root system, which adapts to soil and environmental conditions as the plant matures.

However, managing this vast amount of data and associated attributes requires sophisticated statistical methods to select superior genotypes based on multiple traits rather than solely on grain yield [27,28]. Therefore, multivariate statistical and specialized biplot methods, such as principal component analysis (PCA) and cluster analysis, are essential because they reduce data dimensionality while preserving variance and group similar observations based on selected characteristics, respectively. These methods help resolve functional trade-offs between early vegetative vigor and mature yield components [27]. Additionally, to address the challenges and limitations of conventional selection indices in handling multi-trait data, Olivoto and Nardino [29] introduced the Multi-Trait Genotype-Ideotype Distance Index (MGIDI). This multivariate index, based on factor analysis, evaluates and ranks genotypes by quantifying their distance from a theoretical “ideal” ideotype, thereby enabling simultaneous selection and favorable improvements of multiple traits. Moreover, numerous studies have successfully employed MGIDI to identify and select superior genotypes based on multiple agronomic and physiological traits across diverse breeding programs [30,31,32,33,34,35,36].

In this context, the specific objectives of this study were to: (1) evaluate an association panel of 116 spring bread wheat (*Triticum aestivum* L.) genotypes (106 international lines and 10 local Egyptian cultivars) under non-stressed conditions, focusing on grain yield per plant as the primary response variable, early seedling root system architecture and shoot traits as key predictors, and grain yield attributes as intermediate mediating traits; (2) analyze phenotypic trade-offs, structural associations, and multivariate groupings (via PCA and HCA) and identify superior multi-trait ideotypes via the MGIDI index; and (3) identify contrasting genotype panels exhibiting deep seminal-root angles (>60°) and shallow seminal-root angles (<30°) at the seedling stage as baseline architectural selection criteria for future multi-environment testing and breeding programs aimed at yield optimization and climate adaptation.

## 2. Results

### 2.1. Variability and Genetic Parameters

The descriptive statistics, ANOVA results, and genetic parameters for grain yield and its associated traits are summarized in Table 1. Significant phenotypic variation was observed across all traits, particularly for GYP (0.61–14.78 g), PH (95.75–167.88 cm), and HD (65.25–138.75 days), indicating substantial genetic diversity within the studied germplasm. Furthermore, ANOVA results revealed highly significant differences (*p* < 0.01) among genotypes for all traits, confirming the presence of considerable genetic variability. High coefficients of determination (R^2^), reaching up to 99.42% for HD, were also noted. Phenotypic coefficient of variation (PCV) values were slightly higher than the GCV values, suggesting a minimal environmental influence under the evaluated conditions. The highest GCV and PCV values were recorded for GYP (64.28% and 65.39%) and NSP (30.18% and 34.85%). Additionally, broad-sense heritability (H^2^) was exceptionally high across all traits, ranging from 74.79% for NTP to 99.81% for HD. This combination of high heritability and GCV indicates that traits such as GYP, NSP, and SI are predominantly under genetic control, making phenotypic selection highly effective.

Table 2 presents the descriptive statistics, ANOVA results, and genetic parameters related to seedling root system architecture and shoot traits. The minimum and maximum values reveal a substantial range of phenotypic variation among the wheat genotypes evaluated at the seedling stage. Specifically, the number of seminal roots (RootN) ranged from 3.56 to 6.00, while root length (RL) varied from 8.08 to 15.61 cm. Notably, the parameters related to root angle exhibited considerable variability; the mean root angle (RA mean) ranged from shallow or moderate (55.10°) to deep (74.53°). Consequently, the seedling deep root angle percentage (DRA) showed a wider range from 32.70% to 88.33%. This remarkable phenotypic plasticity, particularly regarding root distribution and angle characteristics, offers a valuable opportunity to select genotypes with optimized root architecture for drought tolerance. The ANOVA analysis revealed highly significant differences (*p* < 0.01) among the wheat genotypes for all assessed root and shoot traits, with R^2^ values ranging from 52.27% (SRA) to 96.29% (GL). Broad-sense heritability (H^2^) was notably high (>70%) for nearly all traits, peaking at 98.09% for GL and 97.52% for ShL, while SRA exhibited moderate heritability at 54.73%. Furthermore, the close alignment between the GCV and PCV values for major traits, including root angle parameters (RA mean and RA median), indicates strong genetic control with minimal environmental influence during the seedling phase under the controlled growth-chamber conditions. The highest GCV values were observed for SRA (38.10%), GL (27.36%), and RaL (26.11%). These findings suggest significant genetic variability and high heritability for key root architecture traits, underscoring the feasibility and effectiveness of early-stage phenotypic selection in developing drought-tolerant wheat genotypes within this germplasm panel.

### 2.2. Data Normality and Distribution

The density plots for the investigated traits reveal distinctly different distribution patterns across the examined germplasm (Figure 1 and Figure 2). The x-axis represents the observed trait values, while the y-axis indicates the probability density. Statistically, the Shapiro–Wilk test classified the traits into two separate groups based on their *p*-values. Significant agronomic and root system architecture (RSA) traits exhibited a strong adherence to a normal distribution (*p* ≥ 0.05), displaying classic symmetrical, bell-shaped curves. This pattern is particularly evident in key root parameters such as mean and median root angles (RA mean: *p* = 0.579; RA median: *p* = 0.470), root length (RL: *p* = 0.507), and biomass traits like shoot fresh weight (SFW: *p* = 0.073). In contrast, traits such as GYP, SRA, and HD significantly deviated from normality (*p* < 0.05). The non-normal distribution of SRA is characterized by pronounced right skewness, indicating a high concentration of genotypes lacking shallow roots. Additionally, HD exhibited a bimodal distribution curve, reflecting a clear separation into early- and late-heading phenological classes, likely influenced by geographical origin and historical selection for maturity (Supplementary Table S2: Normality distribution of studied traits).

### 2.3. Relationships Among Agronomic and Root Traits

The Pearson correlation matrix revealed highly significant and biologically relevant associations among traits related to yield, seedling shoot characteristics, and root system architecture (RSA) traits (Figure 3). Grain yield per plant (GYP) exhibited a strong and highly significant positive correlation (*p* < 0.001) with its attributes (NSP, SPS, SI, and SPAD) and a strong negative association with HD. Additionally, RSR was significantly and positively correlated with GYP (r = 0.33, *p* < 0.001) and SI (r = 0.32, *p* < 0.001). Regarding seedling traits, root length (RL) and seedling shoot length (ShL) showed highly significant positive correlations (*p* < 0.001) with adult plant height (PH), radicle length (RaL), and fresh/dry biomass of shoots (SFW, SDW). In contrast, RL and ShL exhibited significant negative correlations with both GYP (r = −0.28, *p* < 0.01 and r = −0.40, *p* < 0.001, respectively) and SI (r = −0.27, *p* < 0.01 and r = −0.40, *p* < 0.001, respectively). For RSA traits, deep root angles (DRA) demonstrated a strong positive correlation (*p* < 0.001) with both mean and median root angles (RA mean and RA median). In contrast, DRA showed a significant and strong negative correlation with intermediate root angle (IRA). Interestingly, RA mean and RA median were negatively associated with chlorophyll content (SPAD) and yield attributes (Supplementary Table S3: Correlation coefficients among studied traits).

### 2.4. Path Coefficient Analysis and Structural Trait Interrelationships with GYP

To partition the phenotypic correlation between RSA-related traits, seedling traits, attributed traits, and grain yield per plant (GYP) into direct and indirect effects, path coefficient analysis (Figure 4), along with its associated structural path framework (Figure 5), was employed. After addressing multicollinearity among the initial 24 traits, 21 independent variables were retained, accounting for 80.4% of the total variance in GYP (R^2^ = 0.804; residual effect = 0.442). The NSP had the most substantial direct positive effect on GYP (direct effect = 0.511, *p* < 0.001), with a relatively minor total indirect contribution (0.093). This suggests that its impact on yield is primarily direct rather than mediated through other traits. Conversely, HD demonstrated the most significant negative direct effect (−0.325, *p* < 0.001). Additionally, Seed Index (SI, 0.187, *p* = 0.034), Spikelets per Spike (SPS, 0.175, *p* = 0.003), and Radicle Length (RaL, −0.126, *p* = 0.019) also exhibited statistically significant direct effects. Among the seminal root architectural traits, Deep Root Angle (DRA) displayed a slight negative direct effect on grain yield (−0.227, *p* = 0.096), while root angle (RA) mean presented a small positive, yet non-significant direct effect (0.183, *p* = 0.198), and RSR showed a positive direct effect (0.142, *p* = 0.082). Importantly, for RA mean, SDW, RL, and PH, the total indirect effect exceeded the direct effect in absolute value and acted in the opposite direction to the total correlation (e.g., RA mean: direct = +0.183, indirect = −0.334, total r = −0.151). This indicates that much of the apparent association of these traits with yield is mediated through, and partially counteracted by, other traits in the model rather than reflecting a straightforward direct relationship. The equivalence between the sum of direct and indirect effects and each trait’s total genotypic correlation with GYP was validated for all 21 traits, reinforcing the internal consistency of the path decomposition following the correction for multicollinearity within this specific testing environment (Supplementary Table S4).

### 2.5. Hierarchical Cluster Analysis (HCA)

In plant breeding programs, evaluating a large number of genotypes (germplasm collections), such as the116 wheat genotypes in our study, across multiple traits simultaneously is inherently complex. Hierarchical Cluster Analysis (HCA), using Euclidean distance and Ward’s minimum variance method, was employed to group the wheat genotypes into homogeneous clusters based on both seedling root system architecture (RSA) and yield traits at maturity. This approach elucidates how early root architecture influences mature agronomic performance. The cluster analysis (Figure 6) classified the 116 wheat genotypes into four distinct clusters (K = 4), each exhibiting unique biological characteristics. Cluster 1 (blue) includes 12 elite lines and cultivars, namely Gemiza11, Giza171, Sakha95, Sakha96, Misr1, Misr3, Misr4, Sids1, Sids12, Sids14, G057, and G106. This cluster stands out as the most superior, achieving the highest average grain yield per plant (GYP = 11.76 g) and the highest seed index (SI = 4.87 g). Additionally, the seedlings in this cluster exhibit the shortest shoot length (ShL = 7.21 cm) and the highest root-to-shoot ratio (RSR = 1.55). Cluster 3 (green) consists of 39 genotypes, including G001, G030, G036, G084, and G090. These lines are the second most productive, with a GYP of 5.93 g and an SI of 3.71 g, and they display a high spike density of 8.15 NSP. Regarding root traits, this group features a balanced and effective root-angle architecture, characterized by a dominant deep-root-angle percentage of 69.20%. Cluster 2 (red) comprises 23 genotypes, such as G020, G024, G029, and G049. This group demonstrates moderate to high productivity (GYP = 5.54 g, SI = 3.63 g) along with the highest number of tillers per plant (NTP = 11.19). The genotypes in this group exhibit a shallow root orientation, with a lower percentage of deep root angle (DRA = 48.45%) and a higher percentage of intermediate root angle (IRA = 50.07%). Cluster 4 (purple) includes 42 genotypes, such asG002, G007, G034, and G038. It is characterized as the least productive group, with very low grain yield (GYP = 3.14 g) and a low seed index (SI = 3.10 g). In contrast, this cluster exhibits the highest early shoot and root vigor, with the longest shoots (ShL = 10.92 cm) and the longest roots (RL = 12.82 cm) (Supplementary Table S5).

### 2.6. Principal Component Analysis (PCA)

Principal component analysis (PCA) effectively reduced the complexity of 25 seedling root system architecture and yield-related traits across 116 wheat genotypes (Figure 7). The PCA explained 36.11% of the total phenotypic variation, with the first two principal components (PC1 and PC2) accounting for 19.95% (eigenvalue = 4.99) and 16.17% (eigenvalue = 4.04) of the total variance, respectively. PC1 primarily differentiated genotypes based on their early seedling shoot biomass in relation to their mature agronomic performance. Specifically, the right side of Figure 7 shows that PC1 is positively and significantly associated with traits related to early vegetative development. The main contributors to PC1 include SFW (12.55%), ShL (12.35%), and SDW (10.65%) (Supplementary Table S6). Conversely, the left side, PC1, is closely linked to mature grain productivity, with GYP (10.02%) and SI (6.51%) as the principal contributors.

PC2 serves as the primary discriminator of root spatial distribution and rooting depth. The negative PC2 area is predominantly characterized by deep rooting indices. The deep root angle percentage (DRA, 20.76%), mean root angle (RA mean, 20.05%), and median root angle (RA median, 20.38%) contribute the most significantly. In contrast, the positive PC2 area is characterized by shallow rooting indices, particularly the intermediate root angle percentage (IRA, 19.92%). In this context, genotypes located in the lower section of the biplot exhibit steeper seminal root system architecture (predominant DRA > 60°), while those in the upper section display a narrower, shallower, and laterally spread seminal root system (IRA and SRA).

Figure 8 presents a PCA biplot with convex hulls that effectively illustrates the clustering of 116 wheat genotypes into four distinct categories based on 25 traits, including morphological characteristics, seedling root system architecture (RSA), and yield-related traits. Cluster 4 (purple hull, far left) represents the high-yielding elite group, such as Sids1, Gemiza11, Misr1, Misr3, Misr4, Sids12, Sakha96, and Giza171. These genotypes are closely associated with GYP, SI, and RSR. Cluster 2 (red hull, far right) includes genotypes with high vegetative vigor but low grain yield, such as G002, G022, G032, G033, G071, G072, and G074. These genotypes exhibit significant vegetative growth during the seedling phase, as indicated by traits like ShL, SFW, and SDW. Cluster 3 (green hull, top center) comprises genotypes characterized by high tillering and shallow roots, such as G010, G006, G008, G012, G015, and G096. These genotypes are associated with increased root angle (IRA) and number of tillers per plant (NTP). Cluster 1 (blue hull, lower center) represents genotypes with seedling deep rooting architecture, including G047, G052, G065, G079, G085, and G087. 

### 2.7. MGIDI Index-Based Selection of Superior Multi-Trait Genotypes

To evaluate and select the tested genotypes based on their comprehensive performance across 25 multiple target traits simultaneously, the Multi-Trait Genotype-Ideotype Distance Index (MGIDI) was employed. Figure 9 presents a circular radar plot of the MGIDI index, clearly illustrating the precise ranking and distinct differentiation between selected and non-selected genotypes among 116 wheat genotypes. The MGIDI analysis revealed significant variation among evaluated genotypes. Overall MGIDI values ranged from5.49 units, with a minimum of 7.22 for genotype G24 and a maximum of 12.71 for genotype G70. Based on the established selection-intensity threshold, a high-performing subset of 17 genotypes (15% selection intensity) was effectively identified by their lower index scores, indicating closer alignment to the multi-trait target ideotype. The MGIDI values for these elite genotypes ranged from 7.22 for the top-ranked genotype G24 to 9.38 for G31. The selected group included: (G24, G36, G20, G106, G27, G15, Sids14, G46, G64, G26, G76, G90, Gemiza11, G54, G93, G84, and G31) (Figure 9 and Supplementary Table S7).

GYP exhibited the highest overall selection differential, increasing from a population mean of 5.45 g to 7.20 g among the selected genotypes, representing an expected genetic gain of 32.30%. This increase was supported by significant improvements in several traits: SI increased by 21.00% (4.35 g vs. 3.59 g), NSP rose by 21.19% (9.17 spikes compared to 7.57), SL increased by 8.45% (13,04 cm vs. 12.02 cm), and NTP improved by 7.15% (11.09 compared to 10.35). Additionally, chlorophyll content (SPAD) showed a modest gain of 1.41% in the selected genotypes. Positive effects on seedling vigor were also observed, including RFW, which increased by 11.85% (291.82 mg compared to 260.90 mg); RDW, which rose by 10.34% (25.43 mg vs. 23.05 mg); and SFW, which increased by 7.94% (219.20 mg compared to 203.07 mg). Furthermore, RL increased by 1.16%, and root number increased by 2.26%, indicating favorable changes. Heading date showed a desirable reduction of 3.81 days, a −3.50% decrease, promoting earliness without the yield penalty typically associated with early heading.

The percentage of shallow seminal root angle (SRA) decreased by 14.17% (from 0.62% to 0.53%), indicating a steeper and more vertical distribution of the seminal root system. This shift is further supported by an increase in the intermediate seminal root angle (IRA) of 1.84% (from 36.55% to 37.22%). Meanwhile, the deep seminal root angle (DRA) remained predominant and largely stable at 62.25%, with a minimal change of −0.93%.

## 3. Discussion

In modern plant breeding programs and considering the current global climate changes affecting crop productivity and growth, the successful development of high-yielding, climate-resistant wheat varieties (*Triticum aestivum* L.) depends heavily on understanding the genetic interactions between early seedling RSA and mature agronomic traits. This study integrates comprehensive genetic parameters, multivariate s statistical analyses, and index-based selection techniques to elucidate the physiological and genetic dynamics governing GYP under multi-trait selection criteria.

### 3.1. Genetic Variability, Heritability, and Selection Efficiency at Early Stages

The presence of extensive phenotypic and genotypic diversity (*p* < 0.01) across the 25 investigated traits, particularly GYP, PH, HD, and root angle parameters, confirms that the 116-genotype panel harbors substantial exploitable genetic variation. It is important to note that these genetic sources originate from diverse environments, including Asia, Europe, Africa, and the USA, which inevitably contributes to observed differences. Several studies have reported significant genotypic variation in RSA and yield traits among genotypes [24,37,38,39,40,41]. Moreover, the extremely high broad-sense heritability, coupled with the close alignment between PCV and GCV percentages across the studied traits (up to 99.8% for HD, and over 60% for nearly all seedling traits), indicates strong genetic control. These high heritability estimates, combined with elevated GCV values for traits such as GYP, NSP, and root angle parameters, strongly suggest robust genetic regulation. Consequently, direct phenotypic selection at early developmental stages is both feasible and reliable for accumulating desirable alleles in wheat breeding programs. Comparable findings have been reported by Adeleke et al. [40], Bigyan et al. [42], Krause et al. [43], and Lan et al. [44]. Regarding seedling-stage characteristics, the moderate to high heritability of root architecture parameters (GL: 98.1%, ShL: 97.5%, and SRA: 54.7%) emphasizes that RSA is predominantly influenced by genetic factors rather than environmental ones, even at the early developmental stages. However, the limited percentage of genotypes exhibiting SRA values below 30° caused this trait to deviate from a normal distribution, increasing experimental error. This resulted in a moderate heritability estimate and, consequently, reduced early selection efficiency. The high heritability and close alignment between GCV and PCV observed in early RSA traits, such as DRA and RSR, confirm their stable genetic control. This supports the use of non-stressed seedling screening as a highly reproducible and robust baseline proxy for intrinsic rooting potential. The same population was independently evaluated for root and leaf traits under drought stress at the seedling stage, providing complementary information on early-stage drought responses without establishing direct associations between these traits and yield performance [45]. These findings endorse the use of high-throughput seedling screening techniques, such as the clear-pot method, which was specifically developed because traits like narrow root angle and increased seminal root number are heritable and easily phenotyped. These traits serve as effective indicators of deeper mature root systems, which are advantageous for breeding drought-tolerant wheat varieties [25].

### 3.2. Trait Distribution and Correlation Structure

The near-normal distributions of RA mean, RA median, RL, and SFW suggest that these traits are controlled by multiple genes and are suitable for classical quantitative genetic predictions. In contrast, the right-skewed distribution of SRA and the bimodal pattern of HD indicate the presence of major-gene segregation or population stratification. A bimodal distribution of HD is typically interpreted as evidence of two genetically distinct maturity groups within a panel, which corresponds to the presence of significant photoperiod and vernalization loci, rather than representing a purely polygenic continuum. Xu et al. [46] found that the distribution of HD and other traits deviated from normality (*p*  <  0.05), suggesting that this frequency distribution exhibited bidirectional transgressive segregation. The correlation pattern indicated that GYP is positively associated with NSP, SPS, SI, and SPAD, while it shows a negative association with HD and RSR. This pattern reflects the traditional wheat yield-component network that has been widely documented in the literature [42,47,48,49]. Additionally, the timing of maturity, whether early or late, modifies this relationship [50,51]. Notably, the mutual positive correlation between DRA and both the mean and median RA contrasts with its negative correlation with IRA. This observation aligns with the established root-angle continuum in wheat, wherein roots with narrower and deeper growth angles are balanced against those that are shallow and spread laterally, which are advantageous for foraging in the topsoil [41,52,53]. The seminal root angle and the number of roots were identified as significant RSA traits relevant to breeding, with a narrower root angle suggested to be beneficial for deeper rooting and improved yield under stress conditions [25,54]. Importantly, the observed negative correlation between RA mean/median and SPAD/yield attributes contradicts the notion that deep roots are invariably advantageous. Instead, it aligns with research indicating that an overemphasis on deep root biomass may hinder shoot development and reduce aboveground productivity in non-limiting environments [41,53,55].

### 3.3. The Balance Between Seedling Vigor and Grain Yield

One of the crucial biological insights revealed by hierarchical clustering analysis (HCA) and principal component analysis (PCA) is the functional trade-off between early vegetative growth vigor and final grain yield. Specifically, genotypes within Cluster 1, including elite cultivars such as Gemiza11, Sids14, Giza171, Sakha95, Sakha96, Misr1, Misr3, and Misr4, achieved the highest grain yield (GYP = 11.76 g) and seed index (SI = 4.87 g), despite exhibiting significantly shorter seedling shoots (ShL = 7.21 cm) and a higher root-to-shoot ratio (RSR = 1.55). The superiority of these local varieties may be attributed to their adaptation to Egyptian conditions, while other genotypes remain under initial evaluation. In contrast, genotypes in Cluster 4 exhibited enhanced early seedling vegetative vigor, characterized by the longest shoot length (ShL = 10.92 cm) and root length (RL = 12.82 cm), but experienced substantial yield penalties (GYP = 3.14 g). This pattern aligns with studies on wheat diversity, where clustering that incorporates both agro-morphological and root characteristics effectively distinguishes high-yielding modern cultivars from more vegetatively robust yet lower-yielding varieties. While the first two principal components of the PCA accounted for 36.11% of the overall variance, this proportion exceeds the random variation threshold (4% per trait across 25 traits) by more than 4.5-fold. This indicates distinct genetic control and physiological independence of the traits assessed during two different developmental phases (7-day seedling assays compared to mature field trials), thereby fully supporting the eight latent factors included in the MGIDI index. Importantly, this two-dimensional ordination demonstrated biological robustness and simplicity, successfully illustrating the fundamental vegetative–yield trade-off along PC1 and differentiating between deep and shallow rooting patterns along PC2. While the functional trade-offs highlight differing clusters with complementary morphological and agronomic traits, the divergence between these clusters serves solely as an initial marker for parental selection. To establish direct genetic evidence regarding their combining ability and heterosis, subsequent hybridization programs that incorporate diallel or line × tester cross designs are necessary. Chen et al. [56] successfully divided184 genotypes into distinct groups based on root characteristics. Similarly, Zhu et al. [17] compared old and new wheat varieties and found that the high-yielding new varieties had fewer branching roots, less lateral spread, and deeper soil penetration. Conversely, older varieties exhibited larger and more branching roots but did not achieve the highest grain yield.

Addressing the multicollinearity among the original 24 candidate traits significantly improved the clarity of the path analysis. In the revised 21-variable model, the direct path coefficients ranged within a biologically reasonable interval (−0.33 to 0.51). This contrasts sharply with the inflated and internally inconsistent coefficients (exceeding |1|) initially obtained when all compositional root-angle classes (SRA, IRA, DRA) and mathematically dependent traits (ShL, RA median) were included simultaneously, a well-recognized consequence of severe multicollinearity in regression-based path models [57]. Furthermore, NSP exhibited the most significant positive direct effect on GYP (direct effect = 0.51), followed by SI (direct effect = 0.19) and SPS (direct effect = 0.17). The substantial direct influence of NSP underscores the importance of yield-component traits, especially spike productivity, in determining grain yield under the evaluated environmental conditions. These findings suggest that selecting genotypes with high NSP could directly enhance yield, whereas the comparatively minor direct effects of root and seedling traits imply that their contributions to GYP may primarily occur through indirect mechanisms. Conversely, the adverse direct effect of HD supports previous heritability-based findings emphasizing the importance of earliness as a selection criterion under the favorable (non-limiting moisture) conditions observed in this study. In contrast, some studies, such as Dorrani-Nejad et al. [58], have shown that early heading, a highly heritable trait, is an appropriate trait for increasing grain yield and reducing water consumption under drought stress and well-watered conditions, respectively.

Path coefficient analysis indicated that RSR exerted a positive direct influence on GYP (direct effect = 0.14), despite showing negative correlations with biomass traits (SFW and SDW). This suggests a potential trade-off between biomass accumulation and grain yield associated with RSR, implying that selecting an increased RSR may not inherently promote early biomass accumulation, even though it could positively contribute to grain yield. Bainsla et al. [59] observed adjustments between yield components and root architecture across varieties, indicating that different genotypes modulate yield through root versus shoot traits. This supports the notion that trait effects depend on genotype and involve allocation trade-offs. Furthermore, the impact of these traits is influenced by both genotype and environmental factors. Excessive early allocation of resources to shoot biomass creates a competitive sink for assimilates, which can lead to canopy overgrowth, premature depletion of water before anthesis, and reduced translocation of non-structural carbohydrates to developing grain spikes [60,61]. Conversely, elite cultivars prioritize enhancing root biomass rather than shoot elongation during the early growth phase (high RSR), thereby optimizing water absorption without compromising assimilate availability during later stages [62,63].

### 3.4. Root System Architecture and Ensuring Yield Components

For root system architecture, mean RA demonstrated the strongest direct effect on GYP (0.183) among all root-related traits evaluated in this study, surpassing RSR, RDW, RL, RootN, and SRA. This finding provides direct quantitative evidence supporting the seminal root architecture- yield hypothesis. Conversely, DRA (−0.227, *p* = 0.096) and RaL (−0.126, *p* = 0.019) displayed negative direct effects on GYP under the well-watered conditions. The marginally negative direct effect of DRA on yield is consistent with the concept of a trade-off between vegetative vigor and yield: genotypes that invest substantially in the development of deep seminal roots may experience a quantifiable, albeit statistically nonsignificant, yield penalty when soil moisture is sufficient. Crucially, this observation does not contradict the drought-adaptation hypothesis associated with DRA. Instead, it reinforces the idea that the physiological benefits of deep rooting, particularly improved access to subsoil moisture, are context-dependent and are expected to become evident primarily during periods of terminal water deficit, a condition not examined in this study (see study limitations).

It is important to highlight that the RA mean and RA median, although both represent the central tendency of the seminal root angle distribution, are not statistically interchangeable. This is because root angle distributions within a genotype frequently exhibit asymmetry, whereby extreme roots (either very shallow or very deep) can skew the arithmetic mean away from the value that best represents the majority of roots. In contrast, the median remains relatively unaffected by such outliers. Consequently, RA mean and RA median are highly collinear in this dataset, with a variance inflation factor (VIF) of approximately 20 for each when included simultaneously. This high collinearity prevented their simultaneous inclusion in the path model. RA mean was retained because it is the more commonly reported metric in the root architecture literature [64,65], whereas the independent direct contribution of RA median to yield could not be assessed in the current analysis. Future research involving larger genotype panels may be better able to distinguish the unique contributions of these two closely related metrics.

Returning to the importance of root-system depth, DRA enables wheat plants to access subsoil moisture during the reproductive and grain-filling phases, contributing to yield increases of up to 59 kg ha^−1^ mm^−1^ [66,67]. Physiologically, a narrower seedling root angle (DRA) promotes a steeper, more vertically oriented root system. In rainfed or moisture-limited environments, this architectural trait is a cornerstone of the “drought escape” strategy for cereal crops like wheat, barley, and rice, enabling plants to access subsoil water during late-season grain filling and maintain spike density [68,69,70,71]. Under terminal environmental stress, this ability to forage for deep water helps to prevent the abortion of florets and spikelets, thereby maintaining a high number of spikes per unit area (NSP) and ultimately contributing to a greater total grain yield [20,72].

Similarly, the finding that the correlation of RA as mean with yield (−0.151) is primarily influenced by a negative indirect pathway, rather than by its own positive but non-significant direct effect, highlights the importance of path decomposition in distinguishing direct genetic influences from trait-mediated relationships. This finding also cautions against interpreting a simple correlation between RA and GYP as evidence of a direct causal trade-off.

### 3.5. Multi-Trait Ideotype Selection via MGIDI Index

Selecting genotypes based on specific traits frequently results in multicollinearity and undesirable changes in associated secondary traits [73,74]. In the current study, the MGIDI addressed these limitations by dividing the 25 correlated traits into eight orthogonal, uncorrelated latent factors, thereby mitigating multicollinearity and reducing adverse indirect selection outcomes [29]. By assessing the distance between each genotype and a multitrait ideal profile, MGIDI identified 17 elite genotypes (14.6%) with MGIDI index values ranging from 7.22 (G024) to 9.38 (G031). MGIDI effectively managed multicollinearity and enabled the selection of top-performing genotypes, notably G024, G036, and G020. These genotypes successfully combined high tillering capacity (NTP) and spike development (NSP, SL, and SI) with maintained photosynthetic capacity (SPAD) and efficient assimilate partitioning toward grain yield and spike fertility. Importantly, this yield advantage was associated with enhanced early root biomass development, with root dry and fresh weights increasing by 10.34% and 11.85%, respectively. A strong early root system promotes soil exploration, structural anchorage, and the uptake of water and nutrients, thereby providing essential physiological support during grain filling following anthesis. As a phenological adaptation, the genotypes selected by MGIDI showed a slight advancement towards earlier heading (HD; −1.06%), potentially providing an escape from terminal heat in Assiut without yield loss. At the same time, MGIDI maintained a pronounced seminal-root profile, with DRA > 60° at 62.79% of the selected genotypes, a 14.17% reduction in SRA, and a 6.44% increase in RL. This persistent steep root system architecture does not necessarily indicate direct subsoil moisture uptake during grain filling; rather, it serves as a reliable phenotypic benchmark during the early stages. These laboratory-evaluated seedling root attributes showed statistical associations with mature yield and its attributes measured under field conditions in Assiut, consistent with recent concepts on ontogenetic plasticity and climate adaptation [26].

Under field conditions in Assiut, these structural root configurations at the seedling stage demonstrate significant environment-specific correlations with mature yield, consistent with contemporary theories of ontogenetic plasticity and climate adaptation [26]. The convergent validation obtained through the three multivariate techniques (HCA, PCA, and MGIDI) significantly strengthens the reliability of the identified elite genotypes, as prominent lines such as G106 and the commercial cultivars Sids14 and Gemiza11 were consistently co-selected within the high-yielding HCA Cluster 1. The concurrent identification of well-established high-yielding commercial checks (Sids14 and Gemiza11) alongside superior accessions provides biological validation for the discriminative ability of the MGIDI index. These findings affirm that the integration of laboratory-assessed early seedling characteristics with field-assessed adult-plant yield components via MGIDI presents an effective approach for identifying climate-adapted, high-yielding wheat genotypes, consistent with recent applications of MGIDI in challenging conditions [75,76].

### 3.6. Limitations and Future Perspectives

This study was conducted under non-limiting soil moisture conditions maintained by regular surface irrigation at a single site (Assiut, Egypt) during one growing season, without applying regulated water deficit treatments in either the field or the seedling growth chamber. Consequently, all conclusions derived from the field regarding genotype performance, trait correlations, and yield potential are specific to the environment under study. Additionally, assertions concerning subsoil moisture uptake, transpiration efficiency, and drought-induced floret abortion are regarded as physiologically plausible hypotheses based on idealized root architecture models, rather than as results empirically confirmed under stress conditions. The early screening enables high-throughput assessment; the seminal root angle, measured in 7-day-old seedlings, reflects constitutive initial architecture. It cannot be automatically extrapolated to represent the depth of mature roots, biomass distribution, or root system activity in field-grown adult plants.

Root system development exhibits significant developmental plasticity, constantly responding to variations in soil structure, nutrient and water gradients, the metabolic demands of the shoot system, and dynamic genotype-by-environment (G × E) interactions. Although the clear-pot technique provides a consistent and highly heritable measure of root angle, the transparent wall partially constrains the natural root trajectory. This limitation is addressed by methods based on central-growth systems, such as the ClearDepth technique [77]. Additionally, 48 h post-imbibition measurements provide insights into hydration kinetics and the initial dynamics of germination. To avoid the influence of seed size and physiological quality on early seedling vigor, subsequent research should involve pre-sorting seeds by consistent mass to focus on genetic RSA traits.

Finally, wheat grown in Upper Egypt is typically exposed to natural seasonal temperature increases during the grain-filling stage (April–May), a well-documented phenomenon known as terminal heat stress [7,78,79]. Moreover, climatic data from Hamada et al. [80] indicate that ambient temperatures in Assiut can reach as high as 41 °C in April, coinciding with the grain-filling phase and thereby contributing to the significant decrease in grain yield. Although this natural seasonal warming may have influenced the observed differences in grain yield and spikelet/floret-related characteristics among genotypes, the current study did not implement any temperature-controlled or delayed-sowing treatments to isolate or quantify this effect. Therefore, multi-environment trials (MET) across managed water-deficit regimes and delayed-sowing heat stress conditions are strongly recommended. Such future studies are essential to account for developmental plasticity and to validate the climate-resilience potential of both the 17 MGIDI-selected elite genotypes and the early root architectural criteria proposed here.

## 4. Materials and Methods

### 4.1. Plant Materials

A total of 116 genotypes of spring bread wheat (*Triticum aestivum* L.) were evaluated in this study through both controlled seedling-stage phenotyping and mature field trials. This panel included 106 accessions and cultivars of spring wheat obtained from the IPK Gatersleben Genebank (http://www.ipk-gatersleben.de/en/genebank/), representing diverse geographical origins: Europe (54), Asia (30), North America (10), South America (9), Australia (1), and two accessions of unknown origin. Additionally, ten Egyptian bread wheat cultivars were included as local checks to enhance the genetic diversity represented in the study. Comprehensive information regarding all 116 genotypes, including their names, pedigrees, and countries of origin, is provided in Supplementary Table S8.

### 4.2. Experimental Set-Up

Two distinct experiments were conducted in this study: a field trial and a laboratory evaluation under non-limiting moisture conditions.

#### 4.2.1. Field Evaluation of the Genotypes

The field experiment was conducted at the Experimental Station of the Agronomy Department, Faculty of Agriculture, Assiut University, Egypt (27°18′ N, 32°40′ E, elevation 62 m, desert climate), during the 2024–2025 wheat growing season (November–May). Sowing occurred on 17 November 2024. The season was characterized by mild winters transitioning into a hot late spring, with temperatures ranging from a mean low of 6 °C in January to a peak high of 34 °C in May. No rainfall was recorded (0.0 mm), and humidity remained extremely high throughout (https://weatherspark.com) (accessed on 10 September 2026). To ensure non-limiting soil moisture, the trial was flood-irrigated according to standard practices at the Experimental Station, Assiut University, receiving six regular irrigations at non-stress intervals. Soil mechanical analysis classified the site as clay loam, consisting of 26.30% sand, 24.30% silt, and 49.40% clay. Chemical analysis indicated a pH of 7.77, 1.71% organic matter, 0.075% total nitrogen, and 1.19% total calcium carbonate. Following local recommendations, basal fertilizers were applied during seedbed preparation at rates of 100 kg of 15.5% P_2_O_5_ and 25 kg of 48% K_2_O per feddan (1 feddan = 4200 m^2^). Nitrogen was applied at a rate of 163 kg of nitrogen (46% N) per feddan, divided into three doses.

This field trial was conducted using a Randomized Complete Block Design (RCBD) with four replications. Each experimental plot measured 6 m^2^ (1.5 m × 4 m). Within each replication, each genotype was sown in a single 1 m row, with an inter-row spacing of 30 cm and an intra-row spacing of 10 cm between plants. Sowing involved placing three grains per hill. To minimize edge effects, the first and last rows of each plot were planted with a local wheat cultivar, and the entire experiment was surrounded by guard rows of the same cultivar. Three weeks after sowing, seedlings were thinned to maintain one healthy and uniform plant per hill.

The following field and agronomic traits were evaluated:

1. Heading Date (HD, days): Measured on a whole-row basis when 50% of the spikes in the genotype row have emerged.
2. Chlorophyll Content Index (SPAD, units): Measured at the heading stage on the flag leaf using a SPAD meter (SPAD-502, Minolta Camera Co., Ltd., Osaka, Japan), calculated as the average of three individual leaf readings.
3. Plant Height (PH, cm): Measured at maturity as the average height of five randomly selected plants per genotype.
4. Number of Tillers per Plant (NTP): Determined at harvest by calculating the average from five plants.
5. Number of Spikes per Plant (NSP): Recorded at harvest as the average number of spikes from five plants.
6. Spike Length (SL, cm): Measured from the base of the spike to the tip, excluding awns, and calculated as the average length of three representative spikes.
7. Number of Spikelets per Spike (SPS): Calculated as the average count from three representative spikes.
8. Grain Yield per Plant (GYP, g): Measured after harvest as the average grain weight of five individual plants.
9. Seed Index (SI, g): Determined as the average weight of 100 grains, measured from three independent grain samples per genotype.

#### 4.2.2. Phenotypic Evaluation of Seedling Seminal Root Architecture Related Traits

Phenotypic evaluations of seminal root system architecture, seedling characteristics, and kernel traits were conducted under controlled growth chamber conditions. The experiment was arranged in a Completely Randomized Design (CRD) with three replications. For the clear-pot assay, each genotype within a replication was represented by four seeds (subsamples) sown in a single clear plastic cup. To avoid pseudoreplication, seedling measurements within each pot were averaged to produce a single value per experimental unit for statistical analysis.

Seminal root angles and seedling traits were evaluated using a non-soil method through a modified clear-pot technique, based on the procedure described by Richard et al. [25]. The experimental setup was as follows:

1. A sheet of filter paper was fitted inside the inner wall of a clear plastic cup.
2. A horizontal blue reference line was drawn on the inner side of the filter paper to serve as a baseline for determining root angles.
3. Four wheat seeds per genotype were placed vertically at equal intervals along the inner circumference (aligned facing North, South, East, and West) in direct contact with the moist filter paper.
4. Distilled water was added to the bottom of each cup to maintain constant moisture in the filter paper through capillary action, without submerging the seeds.
5. The cups were placed in a growth chamber calibrated to 23 ± 1 °C with a 10 h daily photoperiod under artificial lighting.

Seven days after sowing, digital photographs of the root systems were taken against the filter paper, ensuring clear visibility of the blue reference line. Image analysis was conducted using ImageJ software (V. 1.54r, 2025) [81], and the following traits were recorded:

1. Root Number (RootN): The total number of primary and seminal roots per seedling.
2. Shoot Length (ShL, cm): The distance measured from the base of the seed to the tip of the longest leaf.
3. Root Length (RL, cm): The distance from the base of the root to the tip of the primary seminal root.
4. Root-to-Shoot Ratio (RSR): Calculated as the ratio of total root length to shoot length.
5. Shoot Fresh Weight (SFW, mg): The fresh mass of the shoot measured immediately after excision.
6. Shoot Dry Weight (SDW, mg): The mass of the shoot after oven-drying it to a constant weight.
7. Root Fresh Weight (RFW, mg): The fresh mass of the intact root system.
8. Root Dry Weight (RDW, mg): The mass of the root system after oven-drying to a constant weight.
9. Mean Root Angle (RA mean, degrees): Calculated as the arithmetic mean of all measured seminal root angles per seedling.
10. Median Root Angle (RA median, degrees): The median root angle measured across all roots of each seedling.
11. Shallow Root Angle Percentage (SRA, %): Defined as the percentage of seminal roots with angles of less than 30° (<30°).
12. Intermediate Root Angle Percentage (IRA, %): Defined as the percentage of seminal roots with an angle ranging from 30° to 60° (30° ≤ angle ≤ 60°).
13. Deep Root Angle Percentage (DRA, %): Defined as the percentage of seminal roots with an angle greater than 60° and up to 90° (>60° to 90°).

For kernel traits, ten grains per genotype were placed in Petri dishes containing distilled water and incubated them in a growth chamber at 23 ± 2 °C for 48 h. At 48 h post-incubation, three healthy, fully turgid, germinated grains per replication were selected as subsamples and measured. The measurements were averaged to assess the following traits related to early germination morphology:

1. Grain Length (GL, cm): The maximum distance measured along the longitudinal axis of the kernel.
2. Grain Width (GW, cm): The maximum distance across the transverse axis of the kernel.
3. Radicle Length (RaL, cm): The length of the newly emerged radicle, measured from its point of origin at the seed coat.

### 4.3. Statistical and Genetic Analyses

#### 4.3.1. Analysis of Variance (ANOVA)

Data collected from the field experiment, which included agronomic yield-related traits and was organized using a randomized complete block design (RCBD), along with data from growth chamber assessment of seedling shoot and root system architecture traits structured in a completely randomized design (CRD), underwent analysis of variance (ANOVA) to determine the significance of variation among the assessed spring wheat accessions and cultivars. The linear statistical models used for the RCBD (with both genotypes and blocks were treated as fixed effects) in the statistical linear model and CRD trials were:

$$\mathrm{RCBD}\;\mathrm{trial}\;\mathrm{model}:\;\;\;\;\;\;\;\;Y_{ij}=\;\mu \;+\;g_{i}\;+\;b_{j\;}+\;e_{ij}$$

$$\mathrm{CRD}\;\mathrm{trial}\;\mathrm{model}:\;\;\;\;\;\;\;\;Y_{ij}=\;\mu \;+\;g_{i}\;+\;e_{ij}$$

where $Y_{ij}$ is the observed value of the i-th genotype in the j-th block, μ is the overall mean, $g_{i}$ is the effect of the i-th genotype, $b_{j}$ is the effect of the j-th block, and $e_{ij}$ is the experimental error.

Before parametric analyses, the Shapiro–Wilk test [82] was performed on all quantitative phenotypic traits to evaluate the null hypothesis that the data follow a normal distribution. For traits that did not meet the normality assumption (*p* < 0.05), appropriate transformations were applied to stabilize variances and normalize distributions before performing ANOVA and path analysis. Since the traits SRA, IRA, and DRA were expressed as percentages, they underwent an arcsine square root transformation according to Gomez and Gomez [83] before the analysis of variance. This transformation was applied to ensure homogeneity of variances and to minimize experimental error caused by the non-normal distribution of percentage data. Because the SRA trait included a significant number of genotypes with zero values, a constant of 0.5 was added to each value before transformation to prevent statistical artifacts arising from the presence of repeated zero values. All analyses of variance were conducted using R software (version 09.0-174, 2026).

#### 4.3.2. Variance Components, Genetic Parameters, and Coefficients of Variation

To assess the characteristics of variability, the components of phenotypic variance ($\sigma_{p}^{2}$), genotypic variance ($\sigma_{g}^{2}$), and environmental variance ($\sigma_{e}^{2}$) were calculated from the expected mean squares presented in the ANOVA table as follows:

$$\sigma_{g}^{2}=\frac{MS_{g}-MS_{e}}{r}\;\;\;\;\;\;\;\;\;\;\;\;\;\sigma_{p}^{2}=\sigma_{g}^{2}+\frac{\sigma_{e}^{2}}{r}\;\;\;\;\;\;\;\;\;\;\sigma_{e}^{2}=\;MS_{e}$$

where $MS_{g}$ is the genotypes mean square, $MS_{e}$ is the error mean square, and r is the number of replications.

To measure the extent of variability relative to the mean, phenotypic and genotypic coefficients of variation (PCV and GCV) were calculated as percentages using the following formulas:

$$\mathrm{GCV}\%=\left(\frac{\sqrt{\sigma_{g}^{2}}}{\mu }\right)\times 100\;\;\;\;\;\;\;\;\;\;\;\;\mathrm{PCV}\%=\left(\frac{\sqrt{\sigma_{p}^{2}}}{\mu }\right)\times 100$$

where μ is the overall genotype mean of the respective trait.

#### 4.3.3. Heritability in the Broad Sense ($h_{b}^{2}$)

The broad-sense heritability ($h_{b}^{2}$) on a genotype-mean basis was calculated to determine the percentage of phenotypic variation attributable to genetic causes, assisting in predicting the response to selection for both agronomic and seedling root traits:

$$h_{b}^{2}(\%)=\left(\frac{\sigma_{g}^{2}}{\sigma_{p}^{2}}\right)\times 100$$

#### 4.3.4. Correlation and Multivariate Analyses

- Correlation Analysis: Pearson’s correlation coefficients (r) were calculated among all evaluated agronomic traits, seedling root architecture traits, and kernel traits using genotype means averaged across all replications. This analysis aimed to determine linear associations and identify potential trade-offs or synergistic effects.
- Path coefficient analysis [84] was conducted on standardized genotype mean values (*n* = 116), with grain yield per plant (GYP) as the dependent variable and 24 agronomic, seedling, and root architectural traits as independent variables. Before model fitting, multicollinearity among predictors was assessed using the Variance Inflation Factor (VIF). Since the percentages of Shallow (SRA), Intermediate (IRA), and Deep (DRA) root angles are compositional variables summing to approximately 100% for each genotype, their simultaneous inclusion caused exact linear dependency (aliased coefficients). Consequently, all three possible two-variable subsets were evaluated. SRA exhibited a relatively independent variation compared to the other two categories (VIF = 1.3–1.6 across configurations), while IRA and DRA showed significant collinearity with each other (VIF = 63.1 and 81.0, respectively, when paired), indicating a strong compensatory relationship between the intermediate and deep root-angle categories in this dataset. Therefore, SRA and DRA were retained, with DRA being the root architectural class of primary interest due to its proposed significance in subsoil moisture capture; IRA was excluded. Additionally, two more variables were eliminated due to severe multicollinearity: Shoot Length (ShL; VIF = 34.7), which is a direct mathematical component of the Root-to-Shoot Ratio (RSR = RL/ShL), and RA median (VIF = 19.9), which is redundant with RA mean as both metrics describe the central tendency of the seminal root angle distribution. RA mean was preserved as the more commonly reported metric in the root architecture literature. The final path model included 21 independent variables (HD, SPAD, PH, NTP, NSP, SL, SPS, SI, SFW, SDW, RootN, RL, RFW, RDW, RSR, GW, GL, RaL, RA mean, SRA, DRA), all demonstrating VIF values below 10 (ranging from 1.30 to 9.96; see Supplementary Table S4). The direct effects (standardized path coefficients) were derived from a standardized multiple regression analysis of GYP based on the 21 selected traits. The indirect effects for each trait through every other trait were computed as the product of their genotypic correlation coefficient and the direct effect of the mediating trait, with the total indirect effect for each trait being the sum of these products. The combination of direct and total indirect effects for each trait precisely reflects its overall genotypic correlation with GYP, thereby confirming the internal consistency of the partitioning. The residual (unexplained) path coefficient was determined using the formula √(1 − R^2^). We acknowledge that the resulting ratio of observations to predictors (116:21, approximately 5.5:1) falls below the conventional guideline of 10:1, which is typically recommended for regression-based path models [57]. Nevertheless, all traits were retained in accordance with the principle outlined by Dewey and Lu [84], which states that a trait should not be excluded from the model solely based on a non-significant total correlation, as its direct effect may still be substantial even if offset by an opposing indirect effect. To clarify the direct and indirect structural dependencies that influence GYP along with shoot and root architectural traits, Path analysis, a form of structural equation modeling (SEM), was conducted. All models were specified, estimated, and evaluated using the *lavaan* package [85].
- Principal Component Analysis (PCA) was conducted to reduce dimensionality and identify the most influential traits contributing to the total variation among the 116 spring wheat genotypes. Components with an eigenvalue greater than 1 were retained.
- Cluster Analysis: Hierarchical cluster analysis was performed using Ward’s minimum variance method based on Euclidean distances to group genotypes into distinct clusters. This approach illustrated genetic diversity and relationships among accessions from different geographic regions. The optimal number of clusters was determined objectively by identifying the largest increase in fusion distance within the dendrogram, corresponding to the point of maximum gain in inter-cluster heterogeneity.

#### 4.3.5. Multi-Trait Genotype-Ideotype Distance Index (MGIDI)

To simultaneously select superior genotypes based on multiple evaluated traits without encountering multicollinearity issues, the MGIDI, developed by Olivoto and Nardino [29], was calculated using the *metan* package in R software [86]. Phenotypic genotype means obtained from replicated trials of 116 wheat genotypes, evaluated across 25 agronomic, seminal root architecture, and seedling traits, served as the input matrix. MGIDI integrates multi-trait information into a single stability-based selection index, identifying genotypes closest to the hypothetical ideotype, characterized by optimal values for all measured traits, such as high grain yield, favorable root angles, and robust seedling vigor.

To position all traits on a dimensionless scale and eliminate biases related to unit magnitude, each trait was rescaled to a range of 0 to 100 (*rX_ij_*) using the following formula:

$$rX_{ij}=\frac{\eta_{nj}-\varphi_{nj}}{\eta_{\sigma j}-\varphi_{\sigma j}}\times \left(X_{ij}-\eta_{\sigma j}\right)+\eta_{nj}$$

where $\eta_{nj}$ and $\varphi_{nj}$ represent the new maximum (100) and minimum (0) values for trait j; $\eta_{oj}$ and $\varphi_{oj}$ represent the original maximum and minimum observed values; and $X_{ij}$ represents the mean phenotypic value of genotype i for trait j. Depending on the breeding target:

- For traits where an increase is desirable (e.g., GYP, NTP, NSP, SPS, SL, SI, SPAD, RootN, RL, RSR, SFW, SDW, RFW, RDW, GL, GW, RaL, RA mean, RA median, IRA, and DRA), $\eta_{nj}=100$ and $\varphi_{nj}=0$.
- For traits where a decrease is desirable (e.g., HD, PH, SRA, and ShL), $\eta_{nj}=0$ and $\varphi_{nj}=100$.

An exploratory factor analysis (FA) using principal component extraction was performed on the rescaled trait matrix. Following Kaiser’s criterion (eigenvalues > 1), eight common factors (FA1 to FA8) were identified. To achieve an orthogonal and easily interpretable factor structure while maximizing loading contrasts, a Varimax rotation was applied. Equal weights (w_j_ = 1) were assigned to all factors to ensure that objective selection pressure was maintained. The ideotype was defined as a theoretical entity that achieves the highest rescaled score of 100 across all eight retained factors (*F_j_* = 100). The MGIDI score for each genotype was calculated based on its Euclidean distance from this ideotype:

$${MGIDI}_{i}=\sqrt{\sum_{j=1}^{f}{\left(F_{ij}-F_{j}\right)}^{2}}$$

where $F_{ij}$ is the factorial score of genotype i on the j-th factor (j=1,2,…,8), and $F_{j}$ is the factor score of the ideotype. Genotypes were ranked in ascending order; lower MGIDI scores designate genotypes positioned closest to the ideotype. A selection intensity (SI) of 15% was imposed, resulting in the selection of the top 17 genotypes (selection fraction = 14.66%). The selection differential ($SD=X_{s}-X_{o}$) and percentage selection differential ($SD\%=\left[SD/X_{o}\right]\times 100$) were determined to assess predicted genetic progress across all evaluated traits.

All statistical and biometric analyses, variance estimates, and multivariate procedures were conducted within the RStudio environment 2026 [87].

## 5. Conclusions

This research demonstrates that the integration of seedling root system architecture with mature agronomic traits establishes a robust framework for wheat breeding. This framework is supported by high heritability and strong genetic control for the evaluated panel under the present experimental conditions, which facilitate efficient early selection. While deep seminal root angles represent a valuable constitutive architectural ideotype for potential subsoil moisture capture under drought conditions, mean seminal root angles exhibited a positive, albeit non-significant, direct effect on grain yield under well-watered conditions. Multivariate analyses (PCA, HCA, and MGIDI) revealed a critical functional trade-off between excessive early vegetative shoot vigor and final yield, confirming that genotypes characterized by a balanced, compact shoot and a high root-to-shoot ratio perform better under Egyptian growing conditions. The MGIDI index provided an effective multi-trait selection platform, successfully identifying 17 elite genotypes—including elite cultivars such as Sids14 and Gemiza11, alongside superior lines like G024, G020, and G106—with an expected genetic gain of +32.3% in grain yield. These genotypes represent highly promising parental lines for future multi-environment trials across managed water-deficit and heat-stressed environments.

## Figures and Tables

**Figure 1:**
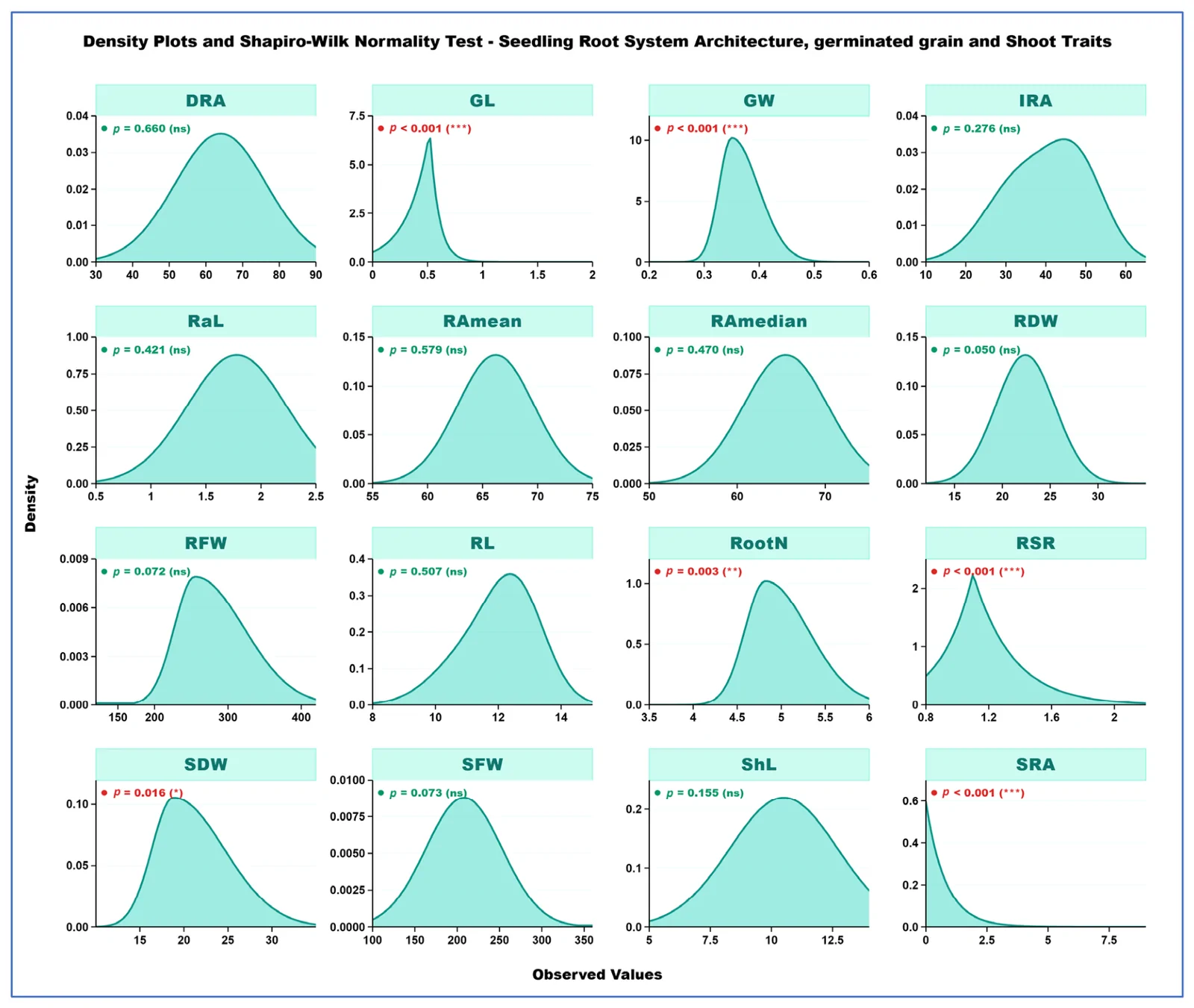
Density plots and Shapiro–Wilk normality tests for seedling root system architecture, germinated grain, and shoot traits. *, ** and *** denote statistical significance at *p* < 0.05, *p* < 0.01 and *p* < 0.001, respectively. NS denotes a non-significant difference at *p* ≥ 0.05.

**Figure 2:**
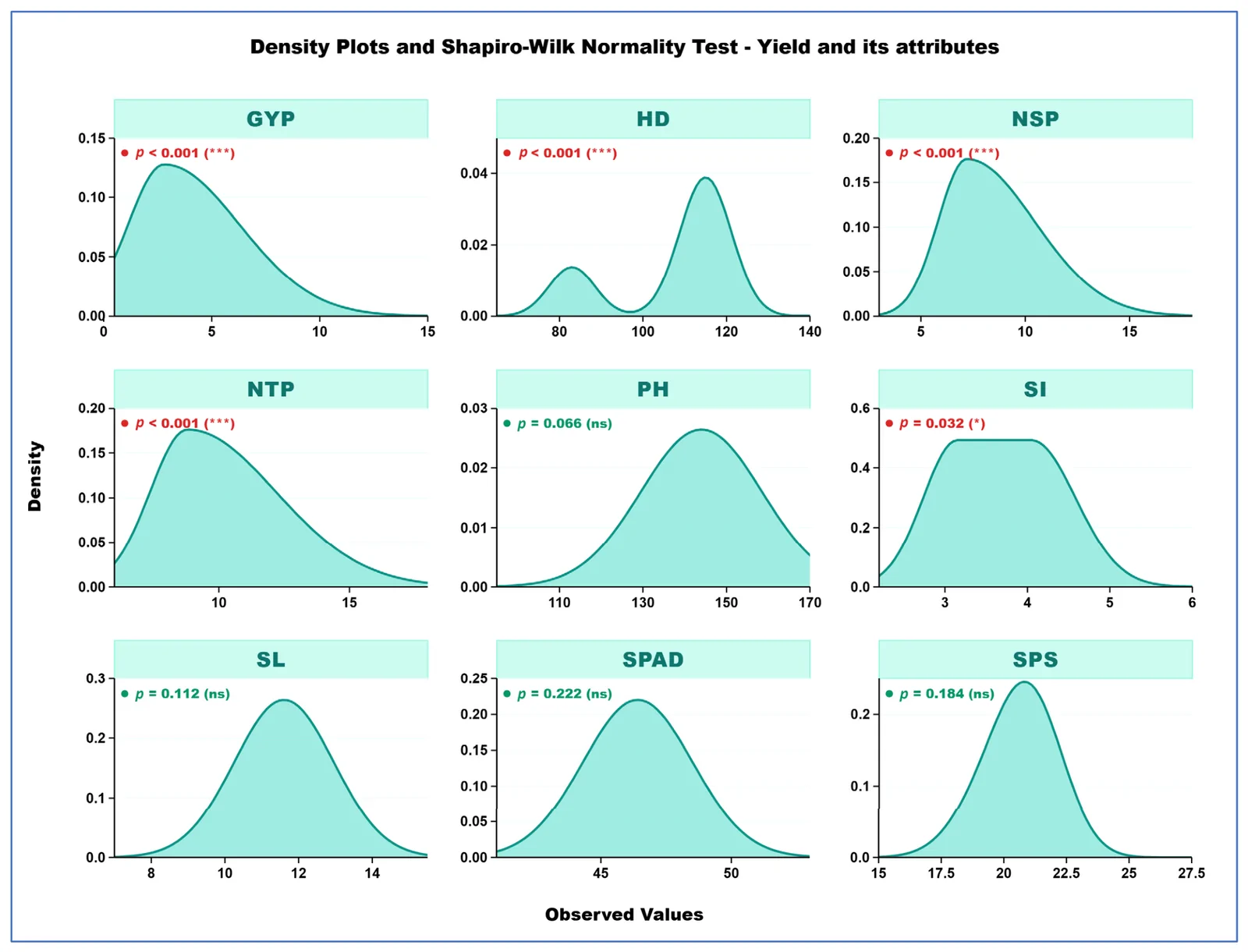
Density plots and Shapiro–Wilk normality test for grain yield and its attributes. * and *** denote statistical significance at *p* < 0.05 and *p* < 0.001, respectively. NS denotes a non-significant difference at *p* ≥ 0.05.

**Figure 3:**
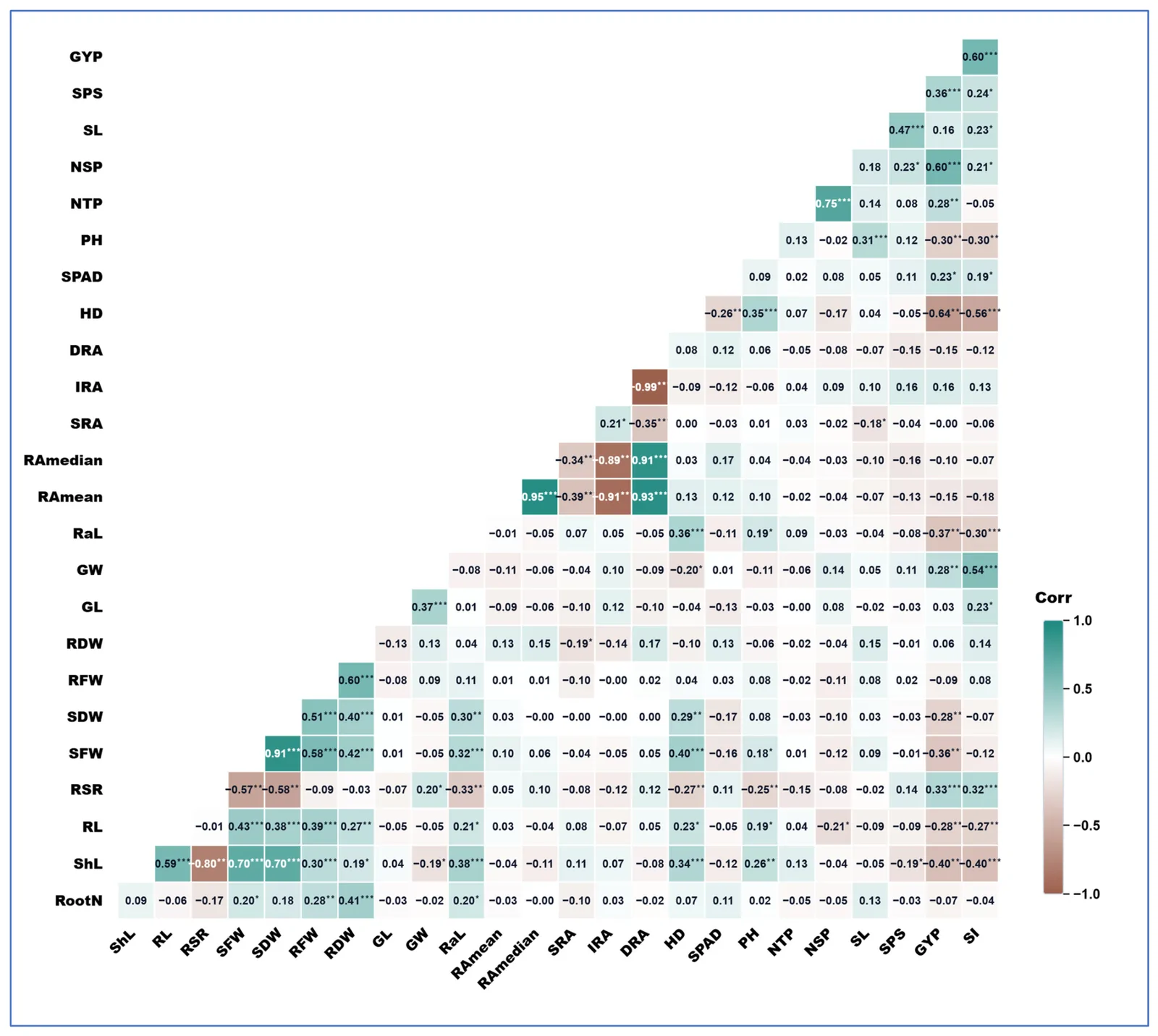
Inter-relationships and phenotypic correlations among agronomic, yield, and root system architecture (RSA) traits. *, ** and *** denote statistical significance at *p* < 0.05, *p* < 0.01 and *p* < 0.001, respectively.

**Figure 4:**
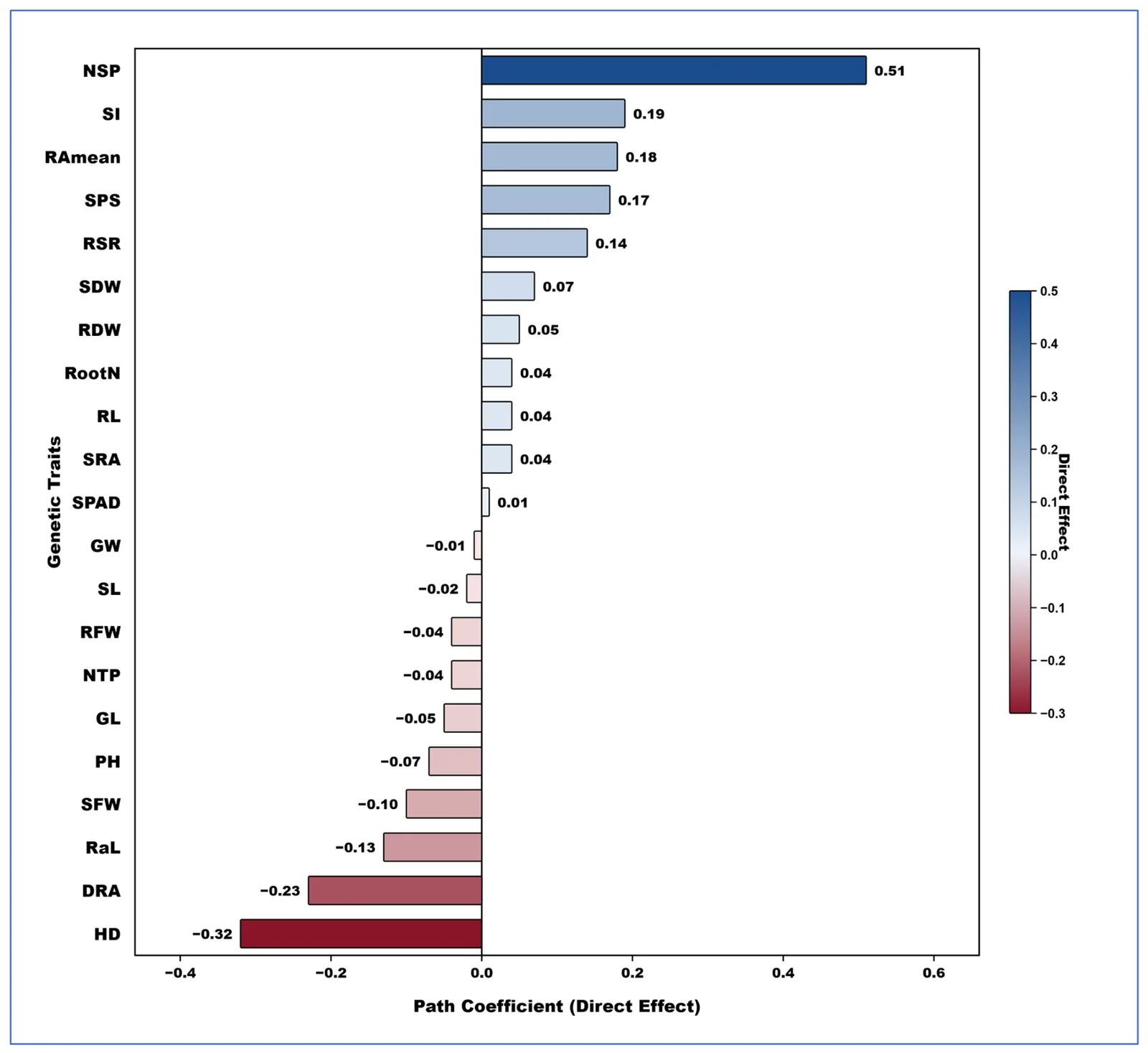
Path coefficient analysis showing the direct effects of genetic seedling (root and shoot) and agronomic traits on GYP in bread wheat.

**Figure 5:**
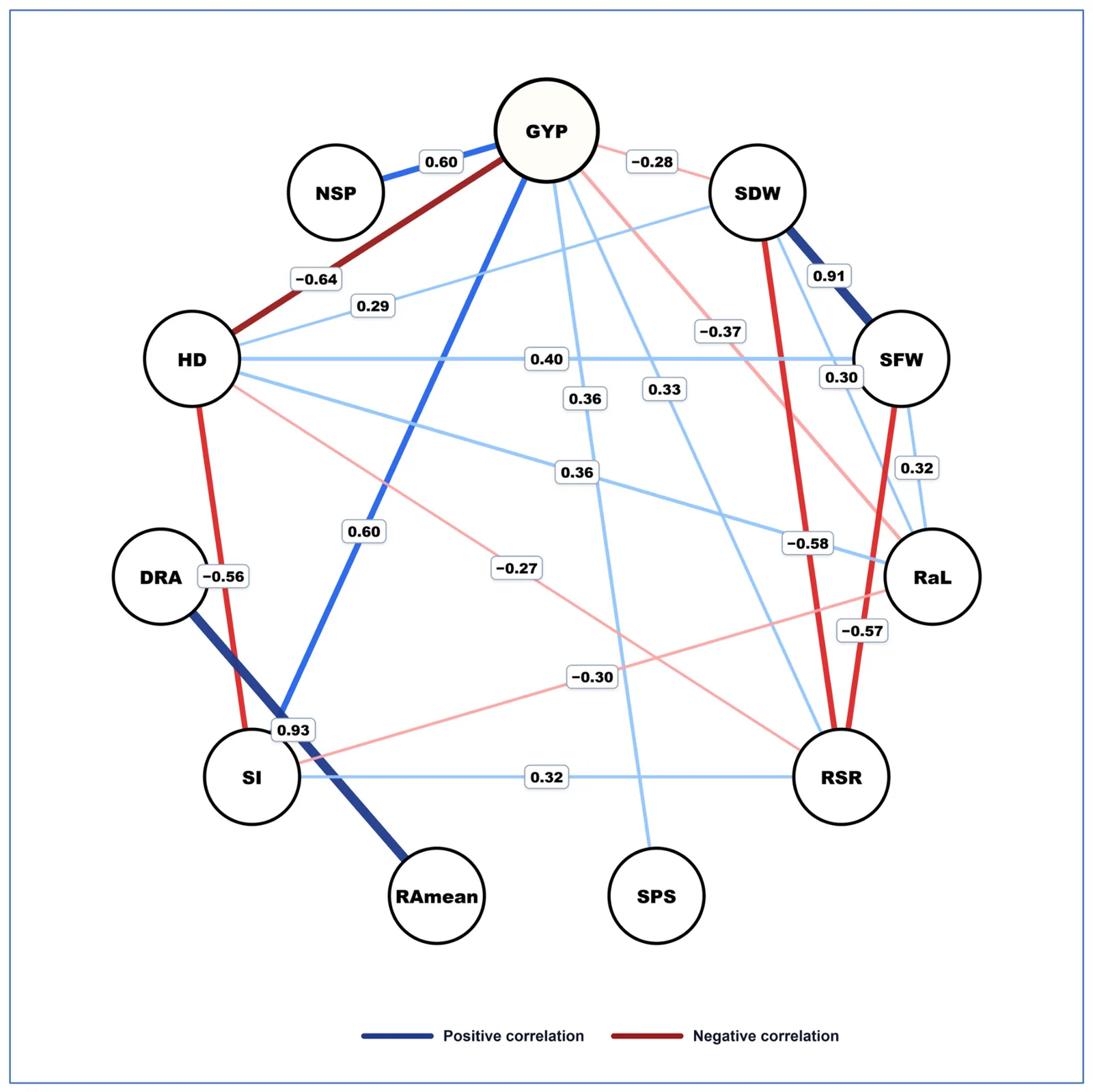
A path analysis network illustrating directional inter-trait dependencies, direct effects, and inter-trait associations among the most influential seedling and agronomic traits related to GYP in bread wheat.

**Figure 6:**
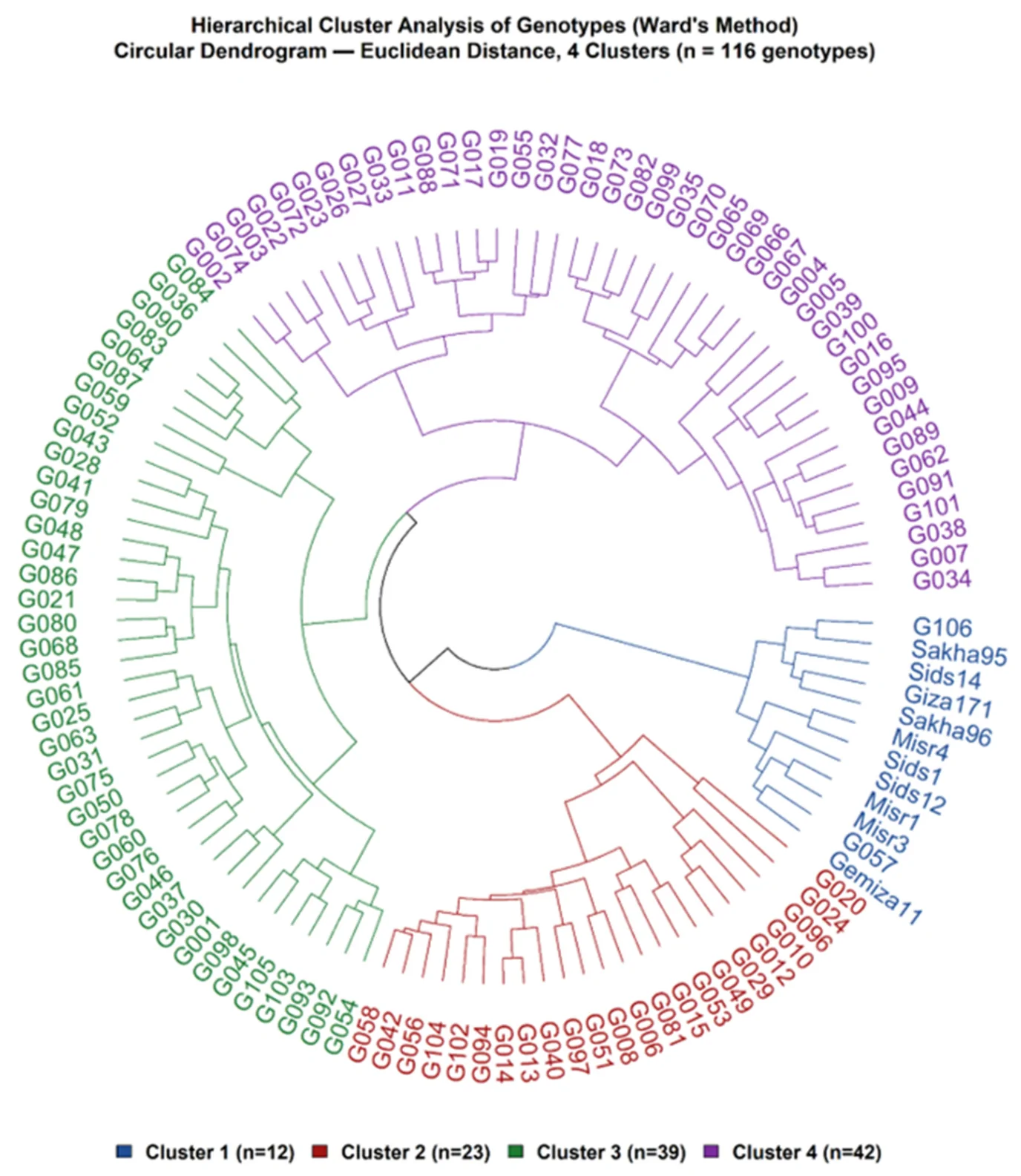
Hierarchical cluster dendrogram of 116 wheat genotypes based on seedling root system architecture (RSA) and grain yield-related attributes using Ward’s minimum variance method.

**Figure 7:**
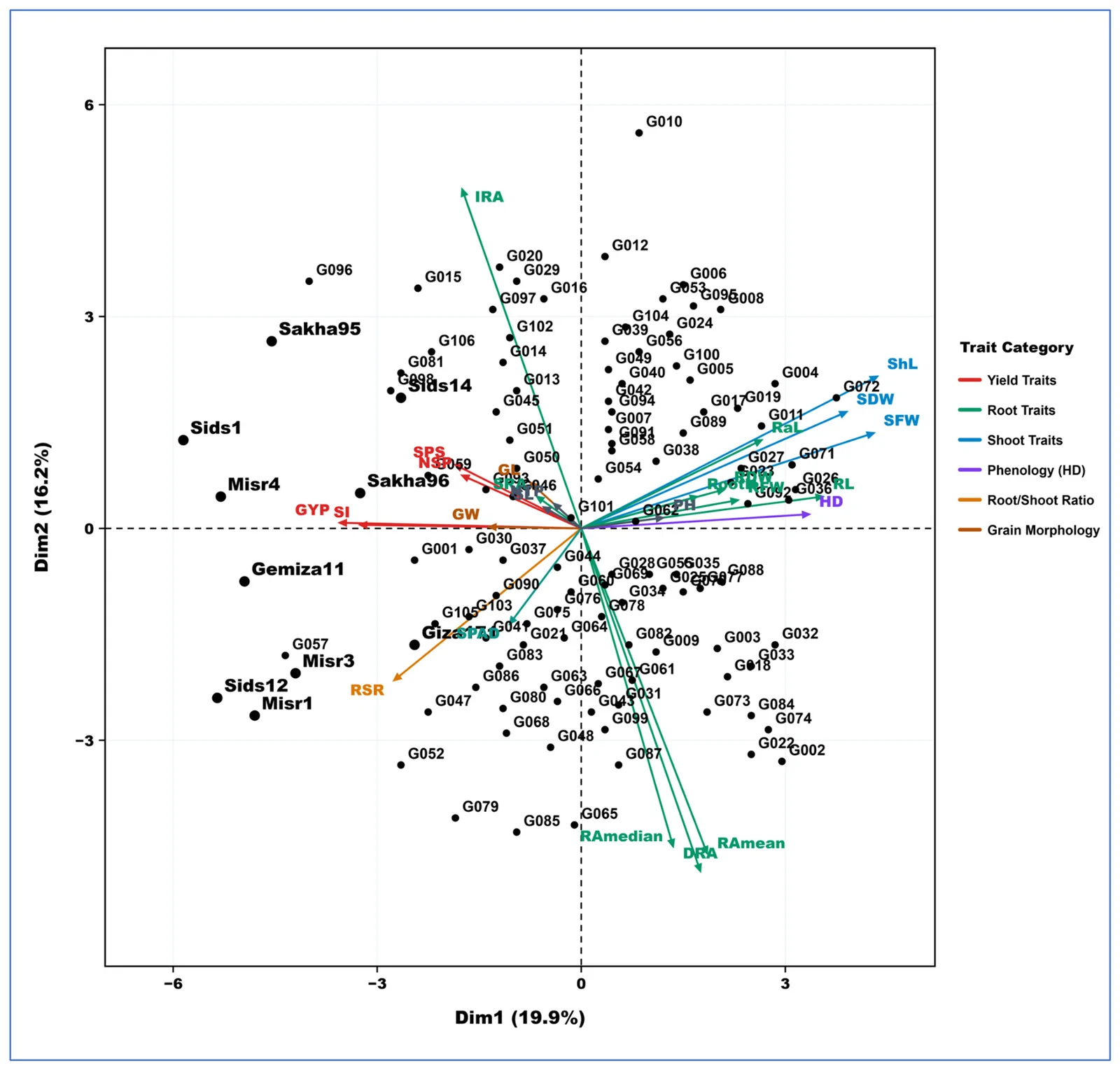
The biplot of Principal Component Analysis (PCA) illustrates the phenotypic ordination of 116 wheat genotypes using 25 traits related to seedling root-system architecture traits, grain yield, and related attributes. The trait vectors are color-coded according to their percentage contribution to the principal components.

**Figure 8:**
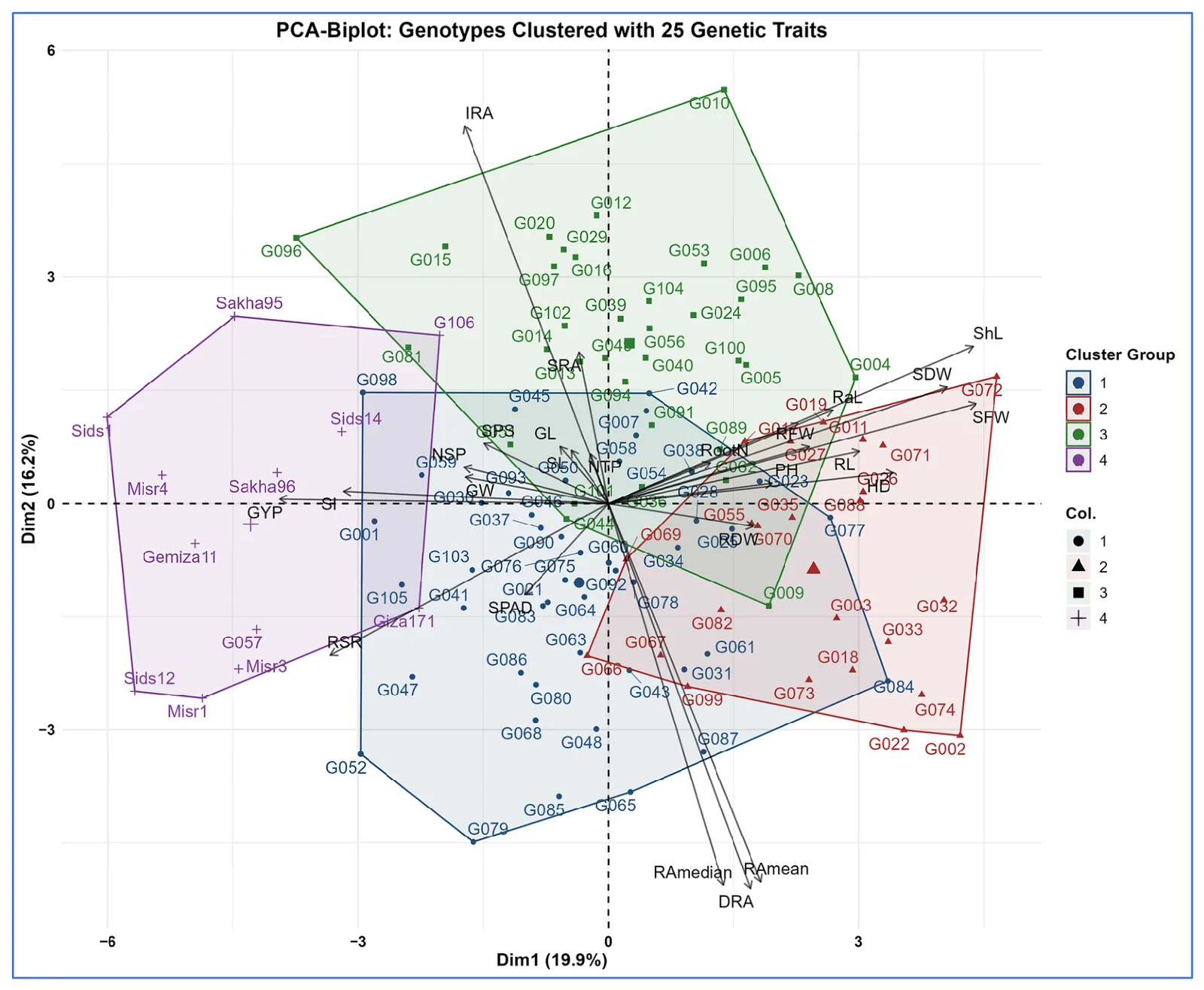
The integrative biplot with convex hulls illustrates the spatial distribution of four clusters and their overlap with seedling root-system architecture traits, grain yield, and related attributes in 116 wheat genotypes.

**Figure 9:**
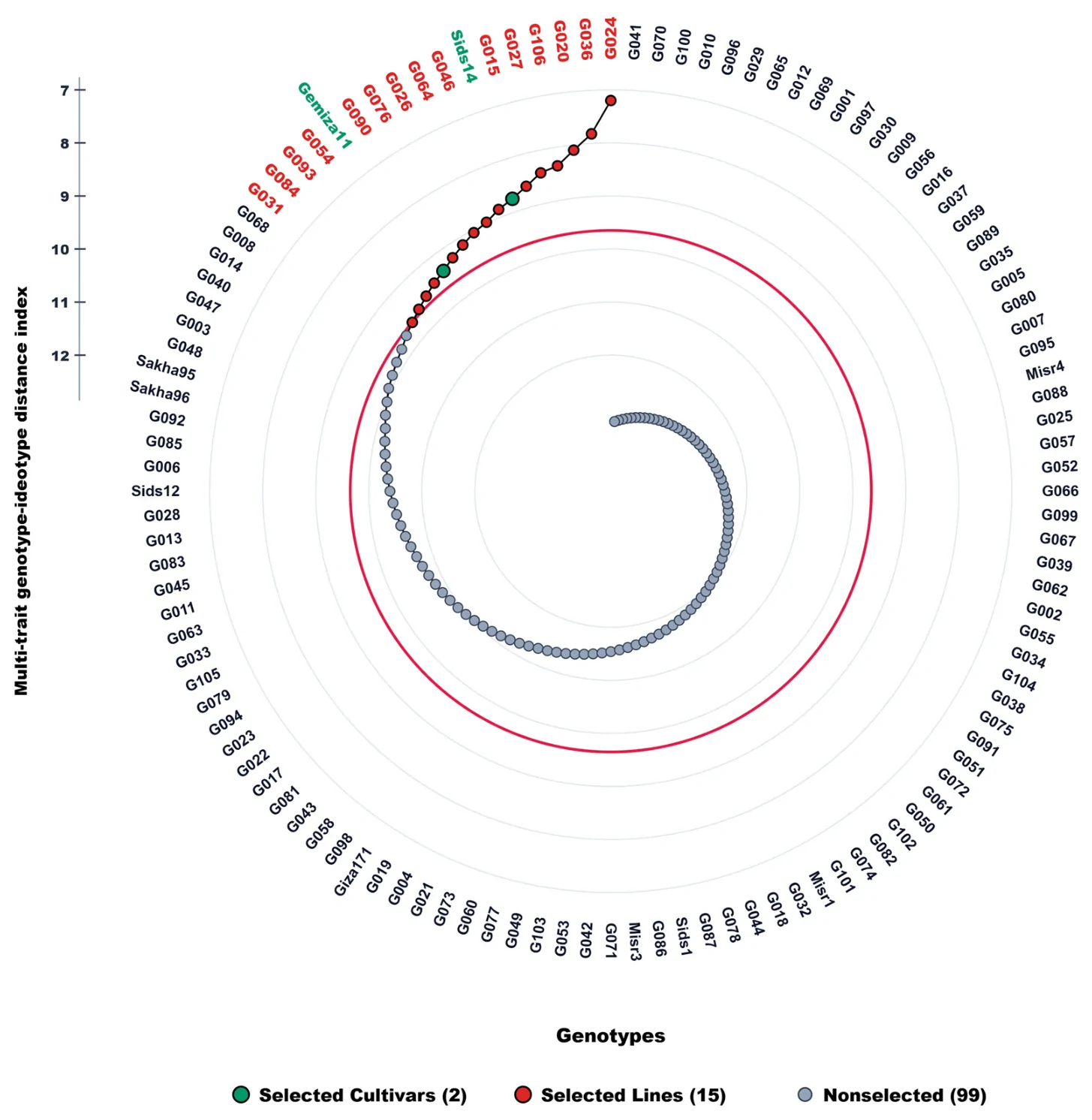
MGIDI index and selection of superior genotypes.

**Table 1:** Descriptive statistics, analysis of variance, and genetic parameters of grain yield and its associated traits in the investigated genotypes.

Trait	Statistics				Mean Squares			Genetic Parameters		
	Mean	Min	Max	R^2^	Block	Genotypes	Error	H^2^%	GCV%	PCV%
HD; days	108.89	65.25	138.75	99.42	110.98 **	935.10 **	1.80	99.81	14.03	14.04
SPAD; units	46.59	41.00	53.39	78.97	203.85 **	24.30 **	2.63	89.18	5.00	5.29
PH; cm	139.54	95.75	167.88	83.67	149.19	871.15 **	56.91	93.47	10.22	10.58
NTP	10.35	6.15	18.70	56.98	1.64	27.26 **	6.87	74.79	21.81	25.22
NSP	7.57	3.35	18.15	57.43	13.10	27.84 **	6.96	74.99	30.18	34.85
SL; cm	12.02	7.05	15.50	63.57	4.05	9.21 **	1.78	80.68	11.34	12.62
SPS	20.72	15.35	27.30	61.38	1.65	16.47 **	3.46	78.97	8.70	9.79
GYP; g	5.45	0.61	14.78	90.83	2.74	50.72 **	1.71	96.63	64.28	65.39
SI; g	3.59	2.28	5.87	96.33	0.09 *	2.29 **	0.03	98.73	20.93	21.07

Where HD is days to 50% heading, SPAD is chlorophyll content, PH is plant height, NTP is the number of tillers per plant, NSP is the number of spikes per plant, SL is spike length, SPS is the number of spikelets per spike, GYP is grain yield per plant, and SI is seed index. Mean, Min, and Max represent the mean, minimum, and maximum values of the traits studied, calculated based on genotype means, respectively (Supplementary Table S1). R^2^, the coefficient of determination, is calculated as the ratio of the genotypic sum of squares to the total sum of squares (SSG/SST). H^2^ is broad-sense heritability. GCV% and PCV% are the genotypic coefficient of variation and phenotypic coefficient of variation, respectively. * and ** denote statistically significant main effects among the 116 genotypes based on the ANOVA F-test at *p* < 0.05 and *p* < 0.01, respectively.

**Table 2:** Descriptive statistics, analysis of variance, and genetic parameters of root system architecture-related traits and shoot traits in the investigated genotypes.

Trait	Statistics				Mean Squares		Genetic Parameters		
	Mean	Min	Max	R^2^	Genotypes	Error	H^2^%	GCV%	PCV%
RN	4.80	3.56	6.00	69.77	0.56 **	0.12	78.52	8.01	9.04
ShL; cm	9.85	5.36	13.78	95.23	10.04 **	0.25	97.52	18.34	18.57
RL; cm	12.04	8.08	15.61	90.31	5.74 **	0.31	94.69	11.18	11.49
RSR	1.25	0.87	2.14	91.89	0.13 **	0.01	95.63	16.22	16.58
SFW, mg	203.07	109.93	354.33	75.34	6410.30 **	1040.19	83.77	20.84	22.76
SDW; mg	20.17	12.43	33.27	70.91	58.35 **	11.86	79.67	19.52	21.87
RFW; mg	260.90	145.00	432.00	73.40	8958.08 **	1609.48	82.03	18.97	20.94
RDW; mg	23.05	13.50	33.73	60.25	39.73 **	12.99	67.30	12.95	15.79
GL; cm	0.55	0.38	1.96	96.29	0.07 **	0.0013	98.09	27.36	27.62
GW; cm	0.35	0.22	0.58	75.25	0.01 **	0.0004	93.49	13.04	13.49
RaL; cm	1.77	0.47	2.76	85.31	0.70 **	0.0596	91.46	26.11	27.30
RA mean; (°)	65.57	55.10	74.53	67.60	48.07 **	11.42	76.24	5.33	6.10
RA median; (°)	65.32	53.34	76.13	67.94	74.75 **	17.48	76.61	6.69	7.64
SRA; %	0.62	0.00	9.05	52.27	0.01 **	0.003	54.73	38.10	51.51
IRA; %	36.55	11.67	62.55	59.87	0.0509 **	0.0169	66.78	16.64	20.37
DRA; %	62.84	32.70	88.33	61.92	0.0546 **	0.0166	69.52	12.17	14.60

Where RN is root number, ShL is shoot length, RL is root length, RSR is root-to-shoot ratio, SFW is shoot fresh weight, SDW is shoot dry weight, RFW is root fresh weight, RDW is root dry weight, GL is grain length, GW is grain width, RaL is radical length, RA mean is mean root angle, RA median is median root angle, SRA is shallow root percentage, IRA is intermediate root percentage, and DRA is deep root angle percentage. Mean, Min, and Max represent the mean, minimum, and maximum values of the trait studied and were calculated based on genotype means, respectively (Supplementary Table S1). R^2^, the coefficient of determination, is calculated as the ratio of the genotypic sum of squares to the total sum of squares (SSG/SST). H^2^ is broad-sense heritability. GCV% and PCV% are the genotypic coefficient of variation and phenotypic coefficient of variation, respectively. ** denote statistically significant main effects among the 116 genotypes based on the ANOVA F-test at *p* < 0.01.

## Data Availability

The original contributions presented in this study are included in the Supplementary Material. Further inquiries can be directed to the corresponding authors.

## Supplementary Materials

The following supporting information can be downloaded at https://www.mdpi.com/article/10.3390/plants15192900/s1: Supplementary Table S1: Genotype means for all studied traits; Supplementary Table S2: Normality distribution of all studied traits; Supplementary Table S3: Correlation Coefficients among studied traits; Supplementary Table S4: Genetic path analysis of investigated traits; Supplementary Table S5: Cluster Analysis of the Studied Traits; Supplementary Table S6: PCA; Supplementary Table S7: MGIDI; Supplementary Table S8: List of wheat lines used in this study.

## Abbreviations

The following abbreviations are used in this manuscript:

#	Abb.	Full Name	Unit/Nature
1	RSA	Root System Architecture	—
2	GYP	Grain Yield per Plant	g
3	PH	Plant Height	cm
4	HD	Heading Date (Days to 50% heading)	days
5	NTP	Number of Tillers per Plant	count
6	NSP	Number of Spikes per Plant	count
7	SL	Spike Length	cm
8	SPS	Number of Spikelets per Spike	count
9	SI	Seed Index (100-grain weight)	g
10	SPAD	Soil–Plant Analysis Development (Chlorophyll Content Index)	SPAD units
11	RootN (RN)	Number of Seminal Roots	count
12	ShL	Shoot Length	cm
13	RL	Root Length	cm
14	RSR	Root-to-Shoot Ratio (Length-based)	ratio (dimensionless)
15	SFW	Shoot Fresh Weight	mg
16	SDW	Shoot Dry Weight	mg
17	RFW	Root Fresh Weight	mg
18	RDW	Root Dry Weight	mg
19	GL	Grain Length	cm
20	GW	Grain Width	cm
21	RaL	Radicle Length	cm
22	RA mean	Mean Seminal Root Angle	° (degrees)
23	RA median	Median Seminal Root Angle	° (degrees)
24	SRA	Shallow Root Angle Percentage (<30°)	%
25	IRA	Intermediate Root Angle Percentage (30–60°)	%
26	DRA	Deep Root Angle Percentage (>60–90°)	%
27	ANOVA	Analysis of Variance	—
28	RCBD	Randomized Complete Block Design	—
29	CRD	Completely Randomized Design	—
30	VIF	Variance Inflation Factor	—
31	SEM	Structural Equation Modeling	—
32	PCV	Phenotypic Coefficient of Variation	%
33	GCV	Genotypic Coefficient of Variation	%
34	H^2^	Broad-Sense Heritability	%
35	R^2^	Coefficient of Determination	%
36	HCA	Hierarchical Cluster Analysis	—
37	PCA	Principal Component Analysis	—
38	PC1/PC2	Principal Component 1/Principal Component 2	% (variance explained)
39	MGIDI	Multi-Trait Genotype-Ideotype Distance Index	index (dimensionless)
40	FA (FA1–FA8)	Factor Analysis (Common Factors 1 to 8)	—
41	SI (Selection)	Selection Intensity	%
42	SD	Selection Differential	original trait units
43	SD%	Percentage Selection Differential (Predicted Genetic Gain)	%
44	G × E	Genotype-by-Environment Interaction	—
45	MET	Multi-Environment Trials	—
46	QTL	Quantitative Trait Loci	—
47	MSg	Mean Square of Genotypes	—
48	MSe	Mean Square of Experimental Error	—
49	SSG	Genotypic Sum of Squares	—
50	SST	Total Sum of Squares	—
